# Comparative phytochemical and biological profiling of *Trifolium rubens* L. leaves, flowers, and callus cultures for cosmetic applications

**DOI:** 10.1038/s41598-026-49836-x

**Published:** 2026-05-15

**Authors:** Marta Klimek-Szczykutowicz, Anna Nowak, Anna Muzykiewicz-Szymańska, Łukasz Kucharski, Paulina Lechwar, Katarzyna Gaweł-Bęben, Karolina Wiśniewska, Renata Piwowarczyk, Adam Kokotkieiwcz, Maria Luczkiewicz, Agnieszka Szopa

**Affiliations:** 1 Department of Pharmaceutical Sciences, Collegium Medicum, Jan Kochanowski University in Kielce, IX Wieków Kielc 19a, Kielce, 25-516 Poland; 2 Department of Cosmetic and Pharmaceutical Chemistry, Pomeranian Medical University in Szczecin, Powstańców Wielkopolskich 72, Szczecin, 70-111 Poland; 3 Department of Cosmetology, University of Information Technology and Management in Rzeszow, Sucharskiego 2, Rzeszow, 35-225 Poland; 4 Department of Environmental Biology, Institute of Biology, Center for Research and Conservation of Biodiversity, Jan Kochanowski University in Kielce, Uniwersytecka 7, Kielce, 25-406 Poland; 5 Chair and Department of Pharmacognosy, Faculty of Pharmacy, Medical University of Gdańsk, J. Hallera 107, Gdańsk, 80-416 Poland; 6 Department of Medicinal Plant and Mushroom Biotechnology, Faculty of Pharmacy, Jagiellonian University Medical College, Medyczna 9, Kraków, 30-688 Poland

**Keywords:** *Trifolium rubens*, plant *in vitro* cultures, Callus cultures, Isoflavonoids, Skin penetration, Biological activity, Biochemistry, Biological techniques, Biotechnology, Drug discovery, Plant sciences

## Abstract

*Trifolium rubens* (Fabaceae) is a rare and regionally endangered European species with limited phytochemical and biological data. This study compares extracts obtained from leaves, flowers, and in vitro derived callus cultures. HPLC–DAD analysis confirmed three major groups of metabolites: flavonoids, isoflavonoids, and phenolic acids, with leaves showing the highest polyphenol levels, including trifolin (1257.13 mg/100gDW) and myricetin (1068.20 mg/100gDW). Callus cultures exhibited a distinct isoflavonoid profile dominated by calycosin-7-*O*-glucoside (516.01 mg/100gDW) and formononetin (103.26 mg/100gDW), accompanied by reduced genistein content compared with parent tissues. All extracts demonstrated antioxidant activity (ABTS IC₅₀: flower 0.02 mg/mL, leaf 0.17 mg/mL, callus 1.51 mg/mL; DPPH IC₅₀: 3.21, 3.93, and 6.16 mg/mL, respectively), moderate inhibition of tyrosinase and elastase, and no cytotoxicity toward HaCaT keratinocytes or A375 melanoma cells. In ex vivo Franz diffusion studies, genistein showed limited permeation but substantial skin accumulation (leaf: 17.07 µg/g; flower: 16.55 µg/g; callus: 2.58 µg/g), supporting localized antioxidant effects. Despite lower total polyphenol content, callus cultures retained relevant bioactivity and accumulated unique isoflavonoids, highlighting their potential as a sustainable alternative source of bioactive compounds. These findings support the cosmetic and dermatological relevance of *T. rubens* and emphasize the importance of optimizing in vitro cultures to enhance metabolite production.

## Introduction

In recent years, interest in cosmetics and dermatological products containing natural ingredients has grown significantly, with plant extracts playing a key role due to their rich content of secondary metabolites that provide antioxidant, antibacterial, anti-inflammatory, and anti-aging effects^1^. Among these compounds, isoflavonoids are particularly noteworthy for their structural similarity to 17-β-estradiol, enabling them to bind to estrogen receptors and influence physiological processes. Genistein (4′,5,7-trihydroxyisoflavone), a prominent isoflavonoid, is widely recognized for its anti-aging properties and potential use in cosmetic formulations^2^. Additionally, in vitro cultivation methods, such as callus cultures, offer a sustainable approach for obtaining these valuable compounds without impacting biodiversity^3^.

The genus *Trifolium* L. (Fabaceae) comprises around 299 species distributed mainly across temperate and subtropical regions, with the greatest diversity in the Mediterranean basin, western North America, and East Africa, while being absent from Southeast Asia and Australia^4^. These species are characterized by a rich content of secondary metabolites, including phenolic acids, flavonoids, isoflavones, saponins, and tannins^5–7^. For centuries, clovers such as *T. pratense* L. and *T. repens* L. have been used both as forage plants and in traditional medicine to treat skin ailments, respiratory conditions, and digestive disorders. Today, their phytoestrogenic isoflavones are valued for antioxidant, anti‑inflammatory, and anticancer properties confirmed by modern studies^8–15^. Extracts from *T. pratense* and *T. repens* are widely incorporated into dietary supplements, teas, and cosmetic formulations due to their anti‑aging and soothing effects, while clover flowers are also used as functional^4,16^.

*Trifolium rubens* L. (ruddy clover) is one of the lesser-studied species of the genus, with limited data on its phytochemical composition and biological activity. This perennial herb, first described by Linnaeus in *Species Plantarum* (1753)^17^, grows mainly in temperate regions of Central and Southern Europe and is characterized by trifoliate leaves, reddish stems up to 90 cm, and dense racemose inflorescences^18^. The native range of this species is central and southern Europe to Ukraine, but is considered rare or endangered in many regions—classified as critically endangered in Bulgaria, Spain, and Luxembourg, and vulnerable in the Czech Republic and Poland^19,20^. *T. rubens* is a regionally endangered species, and its limited natural availability restricts both research and potential applications. Plant biotechnology provides a powerful and ethically justified alternative for studying and utilizing such taxa. In vitro systems, including callus cultures, enable the sustainable production of biologically active metabolites without harvesting wild populations, thereby supporting biodiversity conservation. Numerous studies demonstrate that tissue cultures of rare or endemic species can accumulate high levels of valuable secondary metabolites and often display biological activity comparable to—or even exceeding—that of parent plants. For threatened species such as *T. rubens*, biotechnological approaches are therefore essential tools for both conservation and controlled metabolite production^21–23^.

The objective of this study was to provide comprehensively characterize of the phytochemical profile and biological activity of *T. rubens* extracts derived from both parent plants and in vitro callus cultures. The investigation included a detailed comparative assessment of polyphenol composition, antioxidant capacity, enzyme-inhibitory effects against tyrosinase and elastase, cytotoxic activity, and the skin penetration and accumulation potential of genistein. A key novelty of this work is first successful establishment and biochemical characterization of callus cultures of this rare and regionally endangered species, highlighting their potential as a sustainable and alternative source of bioactive compounds for cosmetic and pharmaceutical use.

## Results

### Callus cultures appearance

**Fig. 1:**
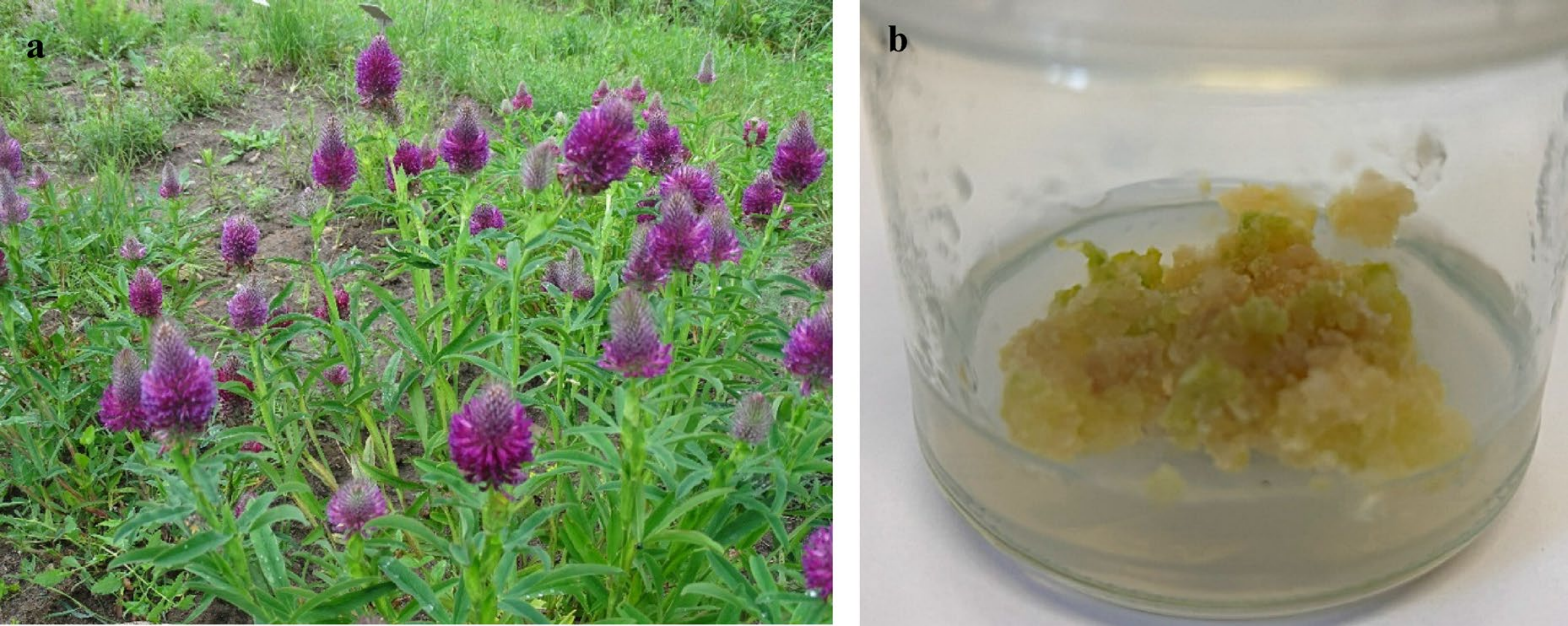
*Trifolium rubens*: **a**-general habit, flower and leaf, **b**- callus cultures after 20 days of growth period cultivated on MS medium containing 1 mg/L BA and 0.5 mg/L NAA and 0.25 mg/L GA_3_.

Callus cultures of *T. rubens* began germinating 30 days after seed sterilization. The medium for initiation and further growth was MS (Murashige and Skoog) medium containing 1 mg/L BA (6-benzyladenine), 0.5 mg/L NAA (1-naphthaleneacetic acid) and 0.25 mg/L GA_3_ (gibberellic acid). Cultures were transferred to a new medium every 20 days. The callus cultures of *T. rubens* were characterized by a green-yellow coloration, soft and friable consistency and homogeneous structure (Fig. 1b). Biomass was harvested after 20 days of growth period.

### Polyphenol profiling by HPLC-DAD

The qualitative and quantitative analyses of the extracts by HPLC-DAD (high-performance liquid chromatography with a diode-array detector) method revealed three major groups of secondary metabolites in *T. rubens*, namely flavonoids, isoflavonoids, and phenolic acids. Among the identified flavonoids were apigenin, kaempferol, myricetin, and trifolin. The highest concentration was confirmed for trifolin, followed by myricetin. Their contents in extracts of *T. rubens* leaf reached 1257.13 and 1068.20 mg/100 g DW (dried weight), respectively, and were significantly higher compared with the extracts from flower and callus culture. Another flavonoid identified in large quantities was kaempferol, which occured in the highest amount in callus culture extracts (396.45 mg/100 g DW), however, without significant statistical differences in comparison to the other extracts originating from plant material. Apigenin was identified only in samples prepared from leaf and flower, while it was not detected in extracts prepared from callus culture. All analyzed extracts were also characterized by the content of isoflavonoids such as: biochanin A, calycosin, calycosin 7-*O*-glucoside, daidzein, genistein, formonoetin and pseudobaptigenin. The isoflavonoid occurring in the largest amount was calycosin 7-*O*-glucoside and it was identified only in callus culture extracts (516.01 mg/100 g DW). The next compound belonging to this group was genistein, whose content ranged from 35.40 mg/100 g DW in callus culture extracts to 193.88 mg/100 g DW in leaf extracts. Callus culture extracts also contained significantly higher amounts of formononetin than all extracts derived from plant material. In the analyzed leaf and flower extracts, 4-feruloylquinic acid was also identified; however, its content was significantly higher in leaf extracts than in flower extracts, reaching 2437.73 and 567.97 mg/100 g DW, respectively. This compound was not detected in extracts prepared from callus cultures (Table 1).

**Table 1:** Qualitative and quantitative (mg/100 g DW ± SD) profiles of phenolic compounds detected in leaf, flower and callus culture of *T. rubens* (*n* = 3; different letters indicate significant differences between the tested extracts; α = 0.05, *nd – not detected).

Group of compounds	Compound	*T. rubens*		
		Leaf	Flower	Callus culture
Flavonoids	Apigenin	66.11 ± 9.84^a^	56.98 ± 13.78^a^	nd*
	Kaempferol	359.11 ± 35.42^ab^	226.28 ± 93.02^a^	396.45 ± 29.26^b^
	Myricetin	1068.20 ± 14.86^b^	237.60 ± 40.76^a^	239.05 ± 1.30^a^
	Trifolin	1257.13 ± 17.88^b^	296.31 ± 29.35^a^	270.13 ± 1.18^a^
Isoflavonoids	Biochanin A	21.38 ± 6.85^a^	24.63 ± 3.64^a^	28.98 ± 1.68^a^
	Calycosin	65.72 ± 3.69^c^	16.64 ± 7.56^a^	37.65 ± 2.63^b^
	Calycosin-7-*O*-glucoside	nd	nd	516.01 ± 1.33
	Daidzein	67.26 ± 6.25^a^	56.54 ± 8.92^a^	111.61 ± 0.96^b^
	Genistein	193.88 ± 17.02^b^	160.82 ± 23.16^b^	35.40 ± 0.71^a^
	Formonoetin	32.82 ± 11.23^a^	29.94 ± 7.20^a^	103.26 ± 10.85^b^
	Pseudobaptigenin	21.38 ± 6.85^a^	13.93 ± 4.50^a^	55.82 ± 0.43^b^
Phenolic acids	4-Feruloylquinic acid	2437.73 ± 92.32^b^	567.97 ± 36.43^a^	nd

### Penetration study

**Fig. 2:**
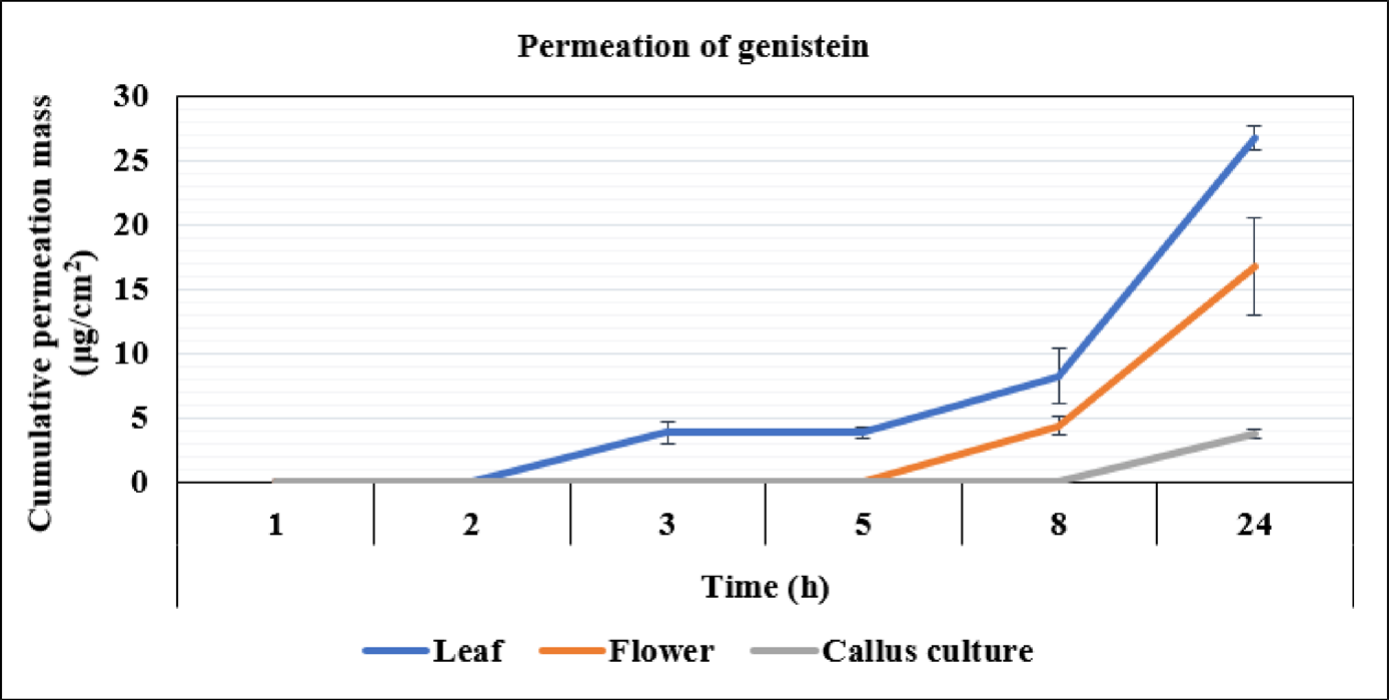
Permeation of genistein from leaf, flower and callus culture extracts of *T. rubens* during a 24-hour study (*n* = 3; different letters indicate significant differences between the tested extracts; α = 0.05).

**Fig.3:**
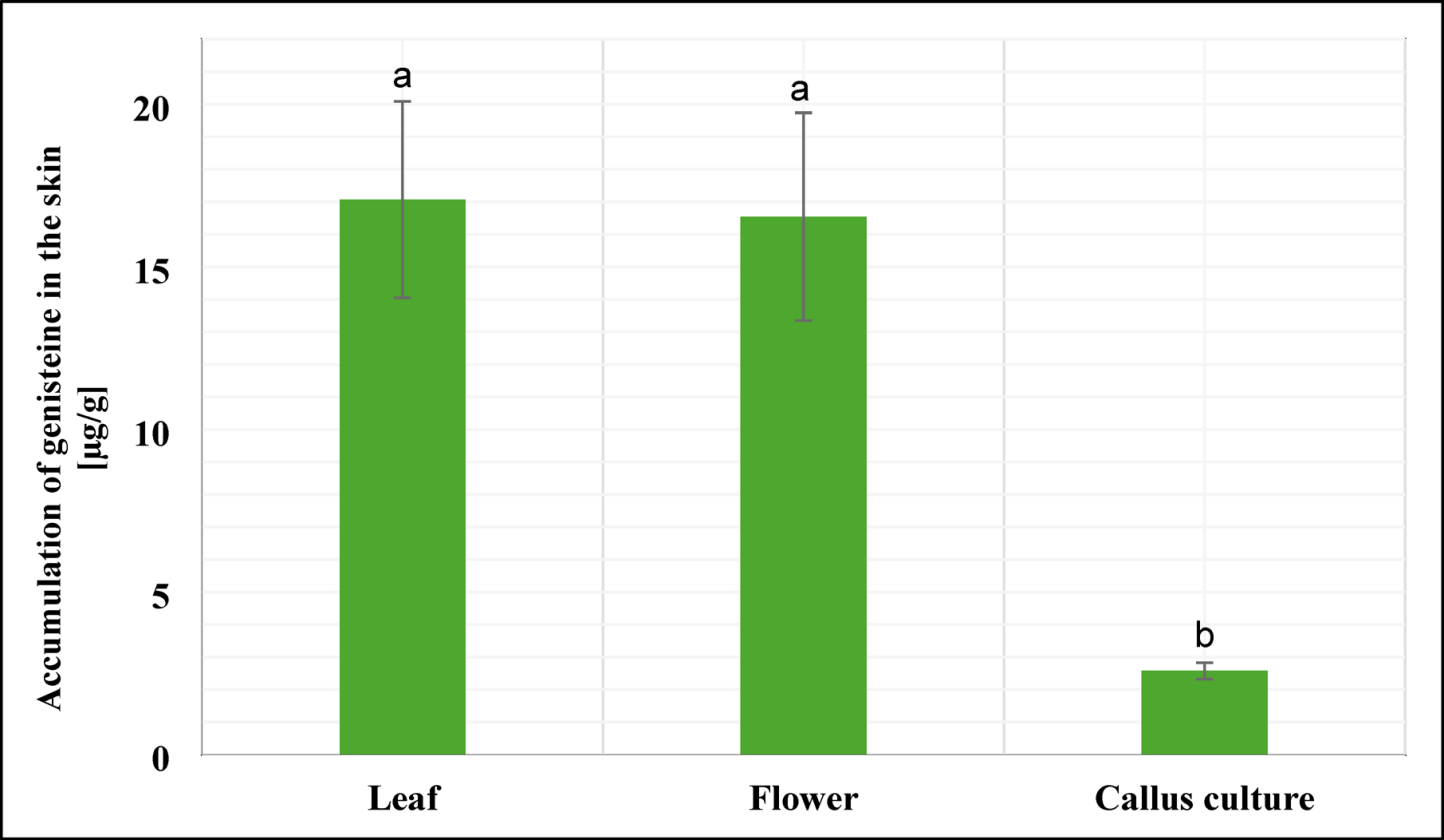
Accumulation in the skin of genistein from leaf, flower, and callus culture of T. rubens extracts during a 24-hour study (n=3; different letters indicate significant differences between the tested extracts; α = 0.05).

The permeation of genistein through porcine skin over 24 h, as assessed using Franz diffusion cells, is presented in Fig. 2, while its accumulation in the skin is shown in Fig. 3. The study was performed for genistein, the main isoflavonoid occurring in *T. rubens*. The highest genistein permeation was observed for leaf extracts, where the compound appeared in the acceptor fluid after 3 h and reached a cumulative mass of 26.73 µg/cm² after 24 h, significantly exceeding the values obtained for flower and callus extracts. In contrast, genistein from flower extracts was first detected at 8 h (16.83 µg/cm² at 24 h). The lowest permeation was recorded for callus culture extracts (3.78 µg/cm²) (Fig. 2).

Genistein accumulated in the skin following treatment with all three extracts (Fig. 3). However, significantly higher levels were observed for leaf and flower extracts (17.07 µg/g and 16.55 µg/g, respectively) compared with callus culture extracts (2.58 µg/g).

**Fig.4:**
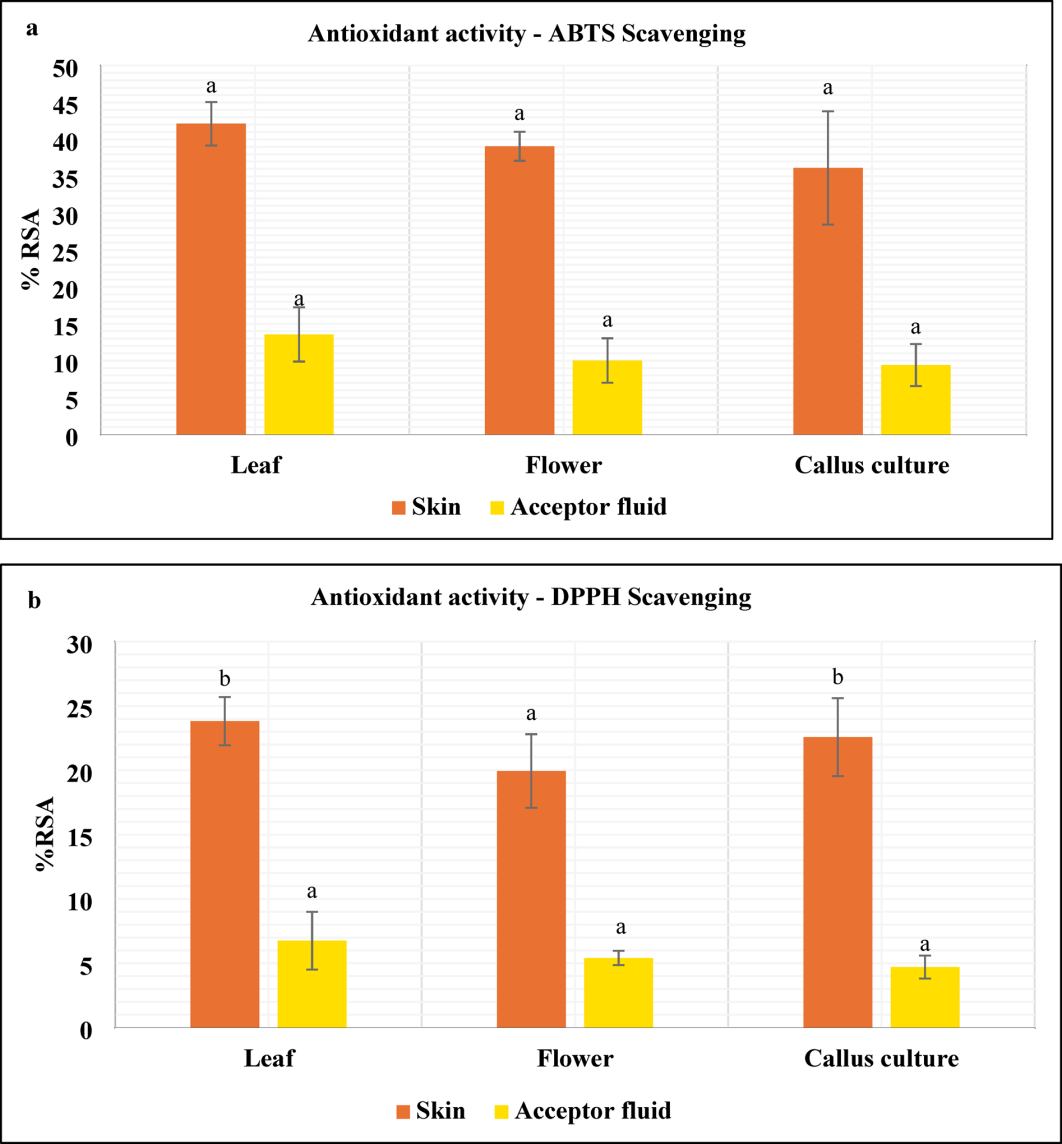
The antioxidant activity of fluid after the skin extraction collected after 24-h penetration (n=3; different letters indicate significant differences between the tested extracts; α = 0.05). **a** – ABTS method, **b** – DPPH method.

Figure 4 presents the antioxidant activity of fluids obtained after the ex vivo permeation experiment. Both the acceptor fluid collected after 24 h and the fluid from skin extraction showed DPPH and ABTS radical-scavenging activity for all extracts tested. In the DPPH assay, the acceptor fluid exhibited %RSA (Radical Scavenging Activity) values of 6.75, 5.41, and 4.70% for leaf, callus, and flower extracts, respectively (Fig. 4b). Similarly, in the ABTS assay, antioxidant activity was highest for leaf extracts (13.56% RSA), followed by flower (10.03% RSA) and callus culture (9.41% RSA) (Fig. 4a). Skin extracts obtained post-study also demonstrated antioxidant activity. In the DPPH assay, the highest %RSA was observed after application of leaf extracts (23.82%), followed by callus (22.58%) and flower extracts (19.94%) (Fig. 4b). The ABTS assay showed a comparable trend, with %RSA values of 42.12, 39.06, and 36.14% for leaf, callus, and flower extracts, respectively (Fig. 4a).

**Fig. 5:**
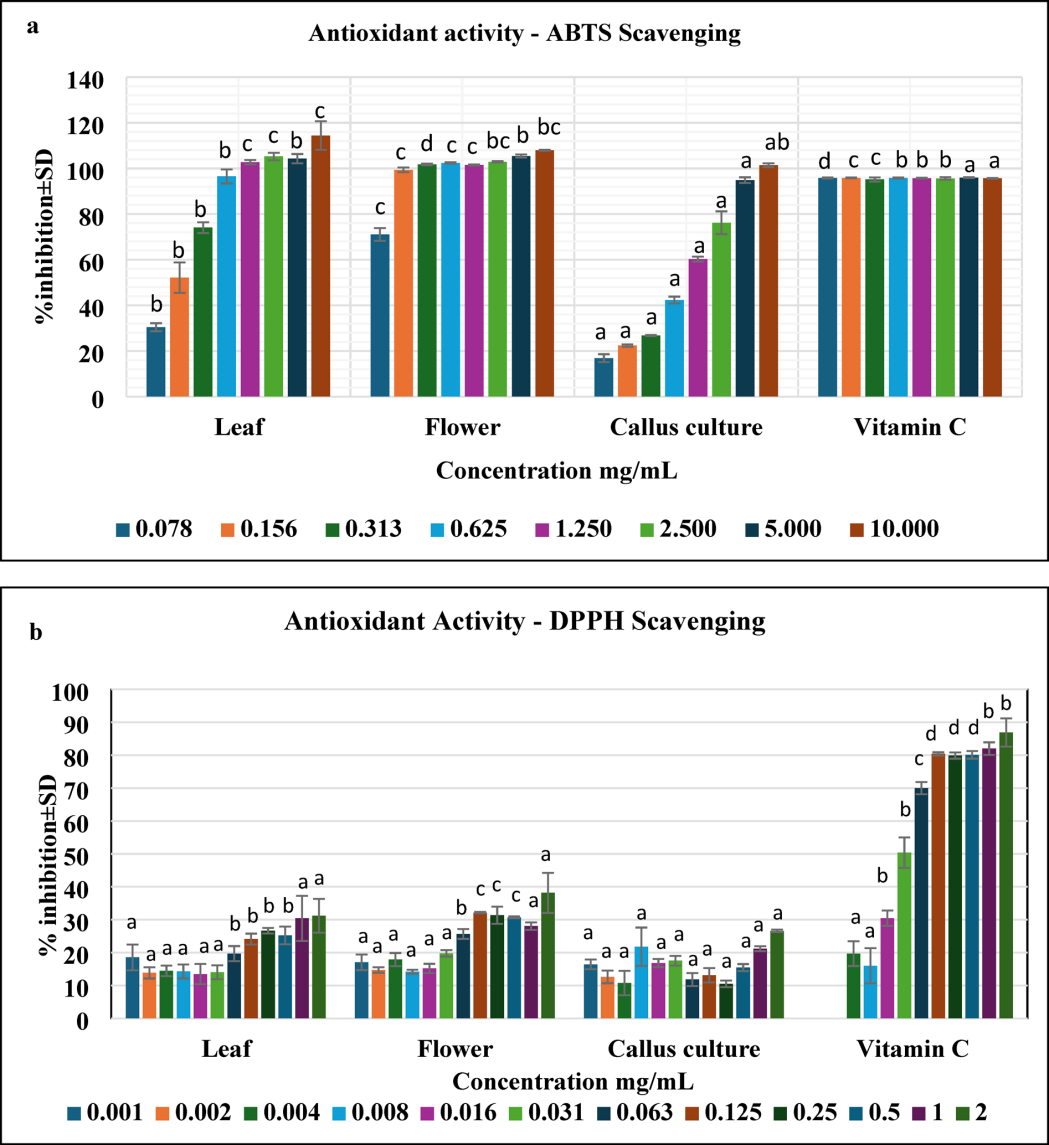
Antioxidant potential (% of oxidation inhibition±SD), for extracts from leaf, flower and callus culture of *T. rubens* (n=3; different letters indicate significant differences between the tested extracts; α = 0.05). **a**- ABTS method, **b**- DPPH method.

## Assessment of biological activity

### The antioxidant potential

The antioxidant capacity of *T. rubens* extracts obtained from leaf, flower, and callus culture was evaluated using ABTS (2,2′-azinobis(3-ethylbenzothiazoline-6-sulfonic acid) and DPPH (2,2-diphenyl-1-picrylhydrazyl) radical scavenging assays (Fig. 5; Table 2). In the ABTS assay, all tested extracts demonstrated notable antioxidant activity — at concentrations of 1.51 mg/mL or higher, each extract was able to neutralize more than 50% of the ABTS radical (Fig. 5a; Table 2). The calculated IC_50_ values indicated that the flower extracts showed the strongest antioxidant effect (IC_50_ = 0.02 mg/mL), followed by the herb extracts (IC_50_ = 0.17 mg/mL). The weakest activity was observed for the callus culture extracts (IC_50_ = 1.51 mg/mL).

The DPPH scavenging assay also confirmed the antioxidant properties of *T. rubens* extracts, although the activity levels were generally lower (Fig. 5b; Table 2). The herb and flower extracts displayed the highest scavenging potential, with IC_50_ values of 3.21 and 3.93 mg/mL, respectively. In contrast, the extracts from callus cultures showed the lowest activity (IC_50_ = 6.16 mg/mL) (Table 2).

**Table 2:** IC₅₀ values (mg/mL) of leaf, flower, and callus culture of *T. rubens* extracts determined by ABTS and DPPH scavenging assays. Results are presented as mean ± SD (*n* = 3).

Assay	*T. rubens*			Vitamin C
	Leaf	Flower	Callus culture	
ABTS	0.17 ± 0.03	0.02 ± 0.01	1.51 ± 0.06	< 0.078
DPPH	3.93 ± 1.24	3.21 ± 1.11	6.16 ± 0.45	0.11 ± 0.06

**Fig. 6:**
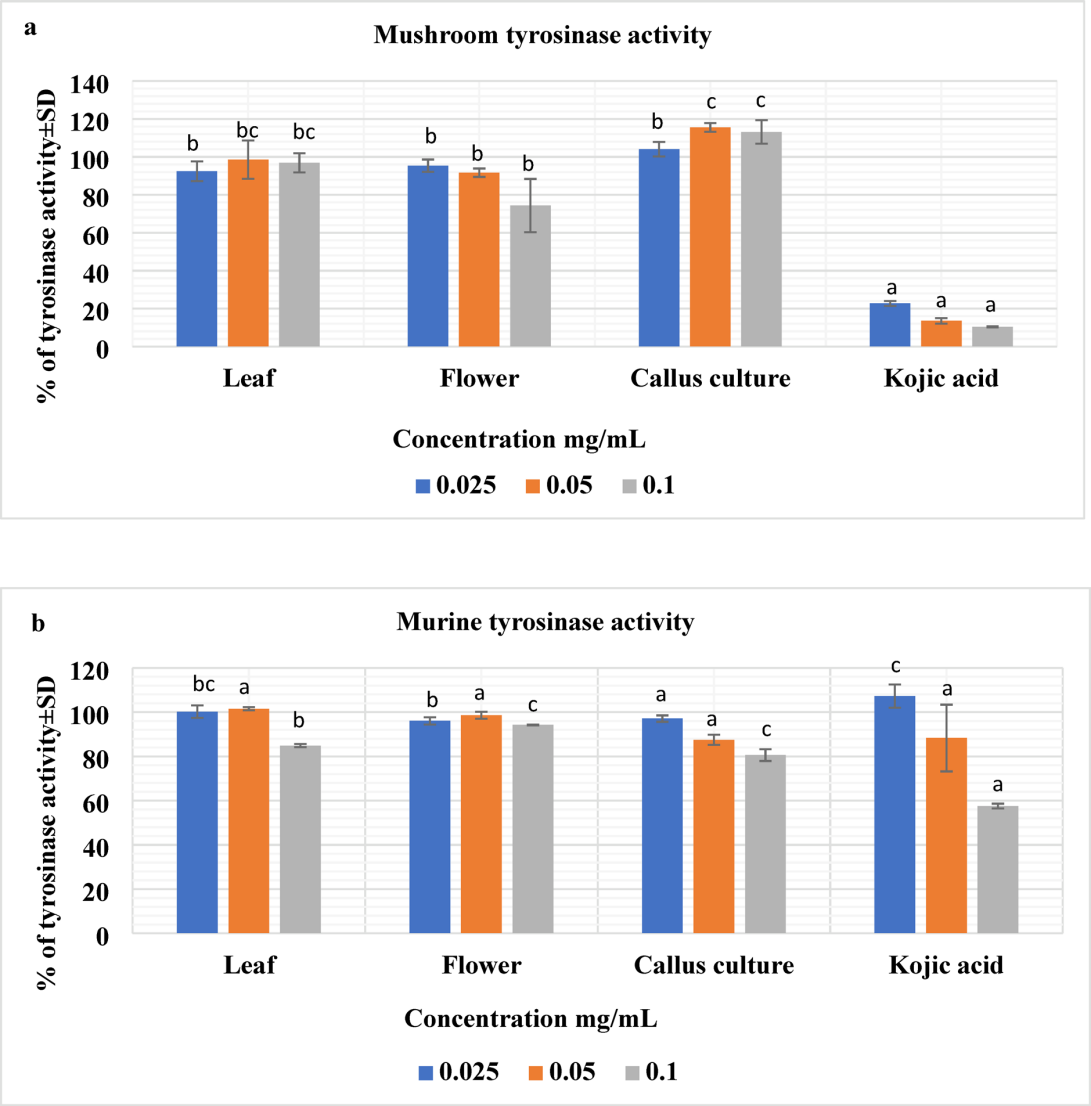
Inhibitory activity of leaf, flower, and callus culture of *T. rubens *extracts on (**a**) mushroom tyrosinase and (**b**) murine tyrosinase, in comparison with kojic acid (n=3; different letters indicate significant differences between the tested extracts; α = 0.05).

### Tyrosinase inhibitory potential

Tyrosinase inhibitory activity was examined using two experimental models: commercially available fungal tyrosinase and murine tyrosinase present in lysates of B16F10 melanoma cells. Owing to the limited availability of purified mammalian tyrosinase, the B16F10 cell lysate serves as a reliable in vitro model for evaluating the skin-lightening potential of synthetic and natural compounds within a mammalian system. As illustrated in Figure 6a, inhibitory activity against fungal tyrosinase was observed exclusively for the flower extracts at a concentration of 0.1 mg/mL, resulting in approximately 25% inhibition. None of the other extracts exhibited inhibitory effects within the tested concentration range (0.1–0.025 mg/mL), and the flower extracts was also inactive at lower concentrations (0.05–0.025 mg/mL).

Figure 6b shows the effect of the tested extracts on murine tyrosinase activity. The highest tyrosinase inhibitory potential was detected for callus cultures and leaf extracts of *T. rubens* (at 0.1 mg/mL) that decreased the tyrosinase activity by 15–20%. Flower extracts, at concentrations ranging from 0.025 to 0.1 mg/mL, did not inhibit murine tyrosinase. Similarly, lower concentrations of leaf and callus culture extracts (0.025–0.05 mg/mL) showed no inhibitory effect on murine tyrosinase activity.

**Fig. 7:**
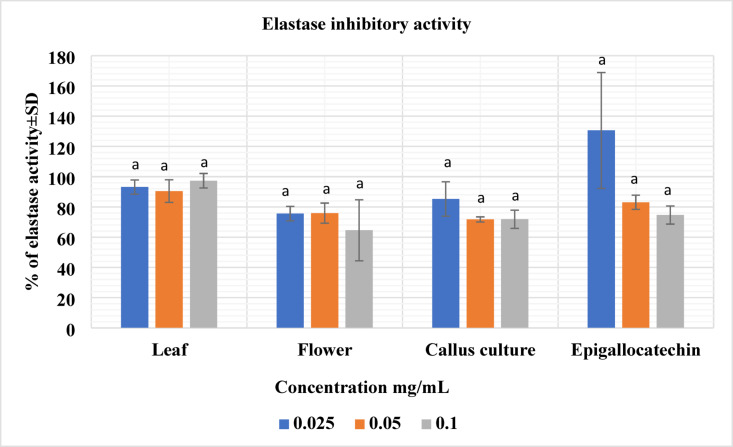
Inhibitory activity of leaf, flower, and callus culture of *T. rubens* extracts on elastase, in comparison with epigallocatechin (n=3; different letters indicate significant differences between the tested extracts; α = 0.05).

### Elastase inhibitory assay

Flower and callus culture extracts showed high potential for inhibiting the elastase enzyme (Fig. 7). In contrast, leaf extracts did not exhibit any inhibitory activity against elastase at the tested concentrations (0.025–0.1 mg/mL). The highest inhibition percentage was obtained for the flower extracts (25–35% inhibition for the concentrations of 0.025–0.1 mg/mL), while the lowest was for extracts from leaf. Furthermore, callus culture extracts exhibited inhibitory activity similar to that of the flower extracts, ranging from 15% to 29% at concentrations of 0.025–0.1 mg/mL (Fig. 7).

**Fig. 8:**
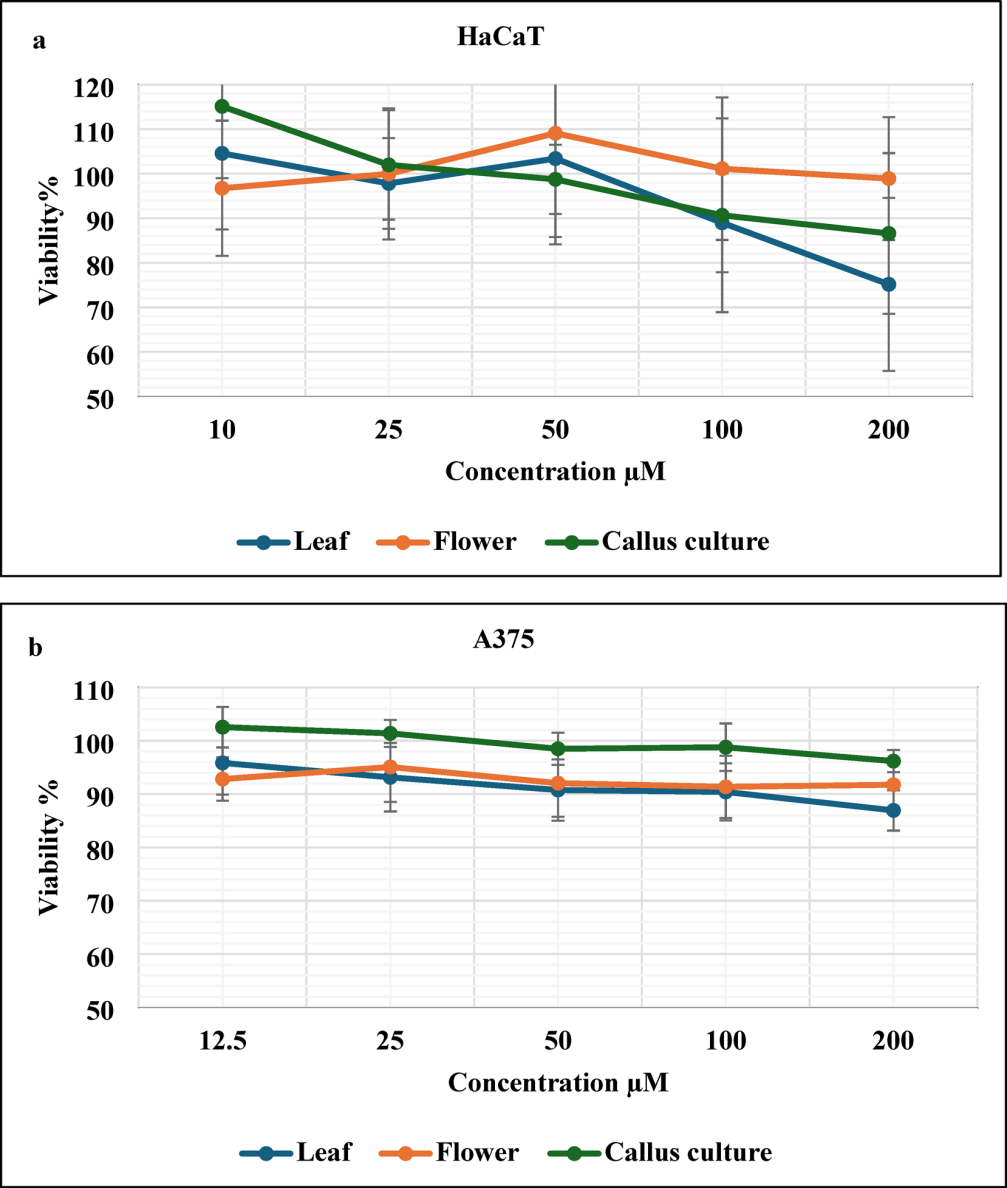
Cytotoxicity of leaf, flower, and callus culture of *T. rubens* extracts against human keratinocytes HaCaT (**a**) and the human malignant melanoma cell line A375 (**b**) following 48 h exposure (n=3; α=0.05).

### In vitro cytotoxicity

The cytotoxicity of leaf, flower, and callus culture extracts of *T. rubens* was assessed to evaluate their safety and potential irritancy. The study was conducted in vitro using human immortalized keratinocytes (HaCaT) (Fig. 8a). All extracts maintained cell viability above 75% across the tested concentrations. Extracts from different plant parts exhibited comparable effects on cell viability, with the lowest values observed for leaf extracts, indicating that the extracts are non-cytotoxic under the experimental conditions.

To evaluate their potential chemopreventive effects, extracts from leaf, flower, and callus cultures of *T. rubens* were tested for cytotoxicity against the human malignant melanoma cell line A375 (Fig. 8b). Experiments were performed at concentrations previously shown to be non-toxic to normal skin cells under the same conditions (Fig. 8a). The results indicated that none of the tested extracts caused a significant reduction in A375 cell viability (Fig. 8b).

**Fig. 9:**
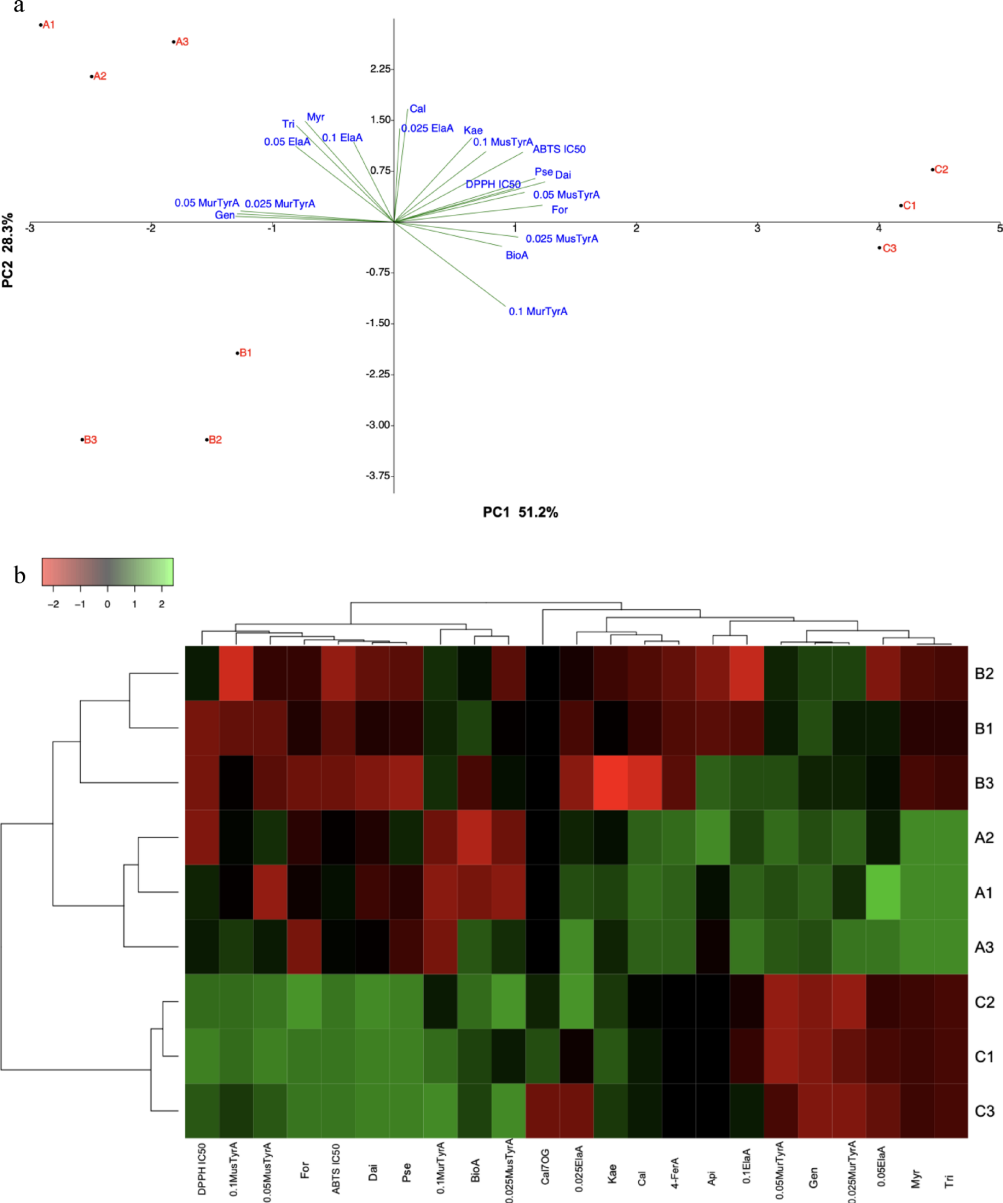
PCA biplot (a) and heatmap (b) showing the multivariate relationships among phenolic compounds and biological activities in leaves (A), flowers (B), and callus cultures (C) of *T. rubens.* Abbreviations: Api – Apigenin (mg/100 g DW), Kae – Kaempferol (mg/100 g DW), Myr – Myricetin (mg/100 g DW), Tri – Trifolin (mg/100 g DW), BioA – Biochanin A (mg/100 g DW), Cal – Calycosin (mg/100 g DW), Cal7OG – Calycosin 7-*O*-glucoside (mg/100 g DW), Dai – Daidzein (mg/100 g DW), Gen – Genistein (mg/100 g DW), For – Formononetin (mg/100 g DW), Pse – Pseudobaptigenin (mg/100 g DW), 4-FerA – 4-Feruloylquinic acid (mg/100 g DW); ABTS IC₅₀ – ABTS radical scavenging activity (mg/mL), DPPH IC₅₀ – DPPH radical scavenging activity (mg/mL); 0.025 MusTyrA, 0.05 MusTyrA, 0.1 MusTyrA – Mushroom tyrosinase activity (%); 0.025 MurTyrA, 0.05 MurTyrA, 0.1 MurTyrA – Murine tyrosinase activity (%); 0.025 ElaA, 0.05 ElaA, 0.1 ElaA – Elastase activity (%).

### PCA and clustering analysis

Principal component analysis (PCA) was conducted to evaluate relationships between the phenolic composition and biological activities of *T. rubens* leaf, flower, and callus culture extracts. The first two principal components explained 79.5% of the total variance, with PC1 accounting for 51.2% and PC2 for 28.3% (Fig. 9). The PCA plot revealed a clear separation of samples according to the type of plant material. Callus culture samples (C1–C3) were clearly separated from leaf (A1–A3) and flower (B1–B3) samples primarily along PC1. In addition to the clear separation between callus cultures and differentiated plant organs, a noticeable differentiation between leaf (A1–A3) and flower (B1–B3) samples was observed, mainly along the PC2 axis. Leaf samples were positioned in the positive PC2 region and showed stronger associations with flavonoids such as myricetin and trifolin, as well as with elastase-related activity, indicating a more pronounced contribution of these compounds to the biological profile of leaf extracts. In contrast, flower samples clustered in the negative PC2 region, reflecting a distinct phenolic composition and weaker correlations with the analyzed enzymatic and antioxidant activities. The wider dispersion of flower samples suggests greater intra-group variability, potentially related to developmental or physiological differences in reproductive tissues. Callus cultures were characterized by a distinct isoflavone-dominated profile, accompanied by specific patterns of tyrosinase inhibition, particularly in murine tyrosinase assays. Overall, PCA demonstrated that both phenolic composition and biological activity profiles were strongly dependent on the type of plant material, with callus cultures exhibiting a clearly distinct metabolic and bioactivity pattern compared to leaf and flower tissues. Although apigenin and 4-feruloylquinic acid (detected in leaves and flowers) as well as calycosin 7-*O*-glucoside (detected exclusively in callus samples) were excluded from PCA due to missing values across sample groups, their distribution patterns further emphasize the pronounced metabolic divergence between callus cultures and differentiated plant organs.

Hierarchical clustering analysis (HCA) combined with heatmap visualization was applied to explore similarities among samples and relationships between phytochemical composition and biological activity (Fig. 9). The heatmap revealed three well-defined and biologically meaningful clusters corresponding to callus cultures (C1–C3), leaf samples (A1–A3), and flower samples (B1–B3), confirming the sample grouping previously observed in PCA. Callus cultures formed a compact and distinct cluster, indicating high internal similarity and a phytochemical profile clearly differentiated from that of leaf and flower tissues. Variables also formed distinct clusters reflecting their chemical and functional relationships. Non-isoflavone phenolic compounds, including flavonoids (apigenin, kaempferol, myricetin, and trifolin) and the phenolic acid 4-feruloylquinic acid, showed greater similarity to each other and were primarily associated with leaf tissues. In contrast, most isoflavones, particularly formononetin, pseudobaptigenin, daidzein, biochanin A, and calycosin derivatives (mainly driven by the callus-specific accumulation of calycosin 7-*O*-glucoside), formed a distinct cluster associated with callus culture samples, whereas genistein was more closely related to leaf and flower tissues. Antioxidant activity parameters (ABTS and DPPH IC₅₀ values) clustered closely and displayed an inverse pattern relative to total phenolic and flavonoid content, indicating stronger antioxidant capacity in samples with higher levels of these compounds. Enzyme-related variables representing residual elastase and mushroom and murine tyrosinase activity (%) clustered together and showed predominantly negative associations with phenolic compounds, confirming that higher phenolic content was linked to stronger enzyme inhibition. Overall, the HCA heatmap highlights clear tissue-specific metabolic signatures and demonstrates a strong relationship between phytochemical composition and biological activity.

## Discussion

This study presents the first comprehensive characterization of the phytochemical composition and biological potential of *T. rubens*. The research focused on the synthesis of polyphenolic compounds as well as the assessment of antioxidant activity and inhibitory effects against elastase and tyrosinase. Extracts obtained from leaf, flower, and callus cultures of *T. rubens* were analyzed. In addition, the permeability of phenolic constituents was investigated, and cytotoxicity assays were performed using HaCaT keratinocytes and A375 malignant melanoma cell lines.

In the present study, callus cultures of *T. rubens* were established and characterized addressing, a significant gap in the existing literature. To the best of our knowledge, research on *T. rubens* has been extremely limited, with only a single report by Cui et al. (1988)^24^, describing somatic embryogenesis and no studies focusing on the phytochemical composition or biological activity of this species. Therefore, the present work represent the first comprehensive investigation of *T. rubens* at both the phytochemical and biological levels, providing a reference framework for future studies on the potential applications of this rare specie. The successful establishment of callus cultures of *T. rubens* is consistent with earlier reports for other represantatives of the genus *Trifolium*, including *T. fragiferum *L.^25^, *T. glomeratum *L.^26^, *T. pratense*^27–29^, *T. resupinatum *L.^30,31^, confirming the general suitability of *Trifolium* species for in vitro cultures conditions. Biotechnological approaches play a key role in the conservation and utilization of rare and endangered plant species, as they enable the evaluation of their chemical profiles and biological potential. Numerous studies have shown that endemic, rare, and threatened species can be successfully propagated under in vitro conditions and may exhibit biological activities comparable to or even exceeding those of their parent plants^32,33^. For example, Türker et al.^34^, in their research on the endemic species *Hypericum bilgehan-bilgilii* Basköse & Savran, reported enhanced physiological performance and increased antioxidant compound levels in plants cultivated in vitro. Their findings highlight the significance of tissue culture as an effective biotechnological strategy for boosting bioactive compound production and suggest that in vitro cultures represent a promising alternative to conventional cultivation methods.

The phytochemical profile of *T. rubens* extracts confirmed the presence of three major groups of secondary metabolites - flavonoids, isoflavones, and phenolic acids. The occurence of flavonoids such as apigenin, kaempferol, myricetin, and trifolin is consistent with earlier reports for leaves and flowers of *T. repens*^35^, *T. pratense*^36^ and *T. incarnatum *L.^37^. Higher flavonoid abundance in aerial plant organs compared with callus cultures has also been described previously for *T. pratense*^28^ indicating that organ differentiation strongly influences flavonoid biosynthesis. Notably callus cultures of *T. rubens* exhibited a distinct flavonoid profile characterized by elevated kaempferol accumulation and the absence of apigenin. The elevated kaempferol content in callus cultures is particularly relevant for cosmetic and dermatological applications, as this compound is known for its antioxidant, anti-inflammatory, and anti-tumor properties^38^. Similar patterns have been reported for *Genista* L. species, where apigenin was detected exclusively in plants grown under natural conditions and not in callus cultures^39^. These findings suggest that in vitro conditions may selectively favor specific branches of the flavonoid biosynthetic pathway.

In addition to flavonoids, *T. rubens* extracts contained a broad spectrum of isoflavonoids, which are key chemotaxonomic markers of the genus *Trifolium*. While the distribution of formononetin, daidzein, and genistein generally agrees with earlier studies of *Trifolium* species^40^, marked quantitative differences were observed relative to previously published data. These differences may be attributed to the application of different extraction methods, variations in plant growth conditions, as well as differences in the drying procedures used prior to extraction. In the study by Dabkevičienė et al.^40^, plant material was dried at 65°C, whereas in our study freeze-drying (lyophilization) was applied, which may have contributed to the higher preservation of thermolabile isoflavones.

A pronounced shift toward isoflavonoid accumulation was observed in callus cultures, which showed the highest total isoflavone content among the tested materials. Similar trends have been reported for *T. pratense* and *Genista* species, where in vitro cultures produced higher concentrations of daidzein and biochanin A, though genistein levels were often lower^39^. In line with these observations, callus cultures of *T. rubens* were characterized by lower genistein content relative to parent plant tissues. Notably, calycosin-7-*O*-glucoside was detected exclusively in callus cultures, highlighting the strong influence of in vitro conditions on isoflavonoid metabolism and emphasizing callus cultures as a distinct source of biologically relevant secondary metabolites^42^.

Isoflavonoids in *T. rubens* are of particular interest due to their strong anti-aging properties^43^. Among them, genistein is widely used in cosmetic formulations for mature skin because of its antioxidant and antiproliferative effects, as well as its ability to protect against UV-induced damage^44^. However, genistein’s lipophilic nature (Log *P* = 3.04) and low water solubility limit its skin penetration, making permeability studies essential for evaluating its potential in topical applications^45^. The present findings demonstrates that, genistein is capable of penetrating the skin from all tested *T. rubens* extracts, with the highest permeation observed for leaf extracts, likely due to its higher genistein concentration and synergistic effects of other metabolites. Differences observed between extracts suggest that penetration efficiency is influenced not only by genistein concentration but also by interactions with co-occurring metabolites. To date, no reports exist on genistein penetration from *Trifolium* extracts, although previous studies have examined pure genistein delivered *via* gels, liposomes, or nanostructured carriers^46^.

From cosmetic perspective, the accumulation of active compounds accumulate within the skin is particulary desirable, as this enables antioxidant effects. Using porcine skin in Franz diffusion cells model, genistein was shown not only to permeate the skin barrier but also to accumulate in skin tissue contributing to measurable antioxidant effects. This observations is highly relevant in the context of skin aging, where excessive generation of reactive oxygen species (ROS) plays a central pathogenic role. The incorporation of natural antioxidants into topical formulations represents an effective strategy to mitigate oxidative stress and genistein is recognized as key contributor to such effects due to its well-documented antioxidant activity^47–49^.

This research provides the first comprehensive evaluation of the antioxidant, tyrosinase-inhibitory, and elastase-inhibitory properties of *T. rubens* extracts obtained from leaf, flower, and callus cultures, and confirms their lack of cytotoxicity toward HaCaT cell lines. The comparative analysis of extracts derived from differentiated plant organs and in vitro cultures highlights the strong influence of tissue type and cultivation conditions on biological activity.

The marked differences observed between assays reflect not only the distinct chemical profiles of the extracts but also the divergent analytical principles of the ABTS and DPPH methods Consistent with previous methodological observations, ABTS generally indicated higher antioxidant capacity across samples, reflecting its sensitivity to both hydrophilic and lipophilic antioxidants, whereas DPPH preferentially detects compounds acting predominantly through hydrogen atom transfer mechanisms.Together, these methodological differences explain why *T. rubens* extracts—characterized by diverse phenolic structures—show assay-dependent variation in their measured antioxidant capacity^50^.

There is a lack of studies investigating the antioxidant potential of *T. rubens* extracts. However, research conducted on other species within the *Trifolium* genus confirms their antioxidant properties. For instance, Esmaeili et al.^28^ demonstrated that aerial parts of *T. pratense* showed higher antioxidant capacity than in vitro and callus cultures. Interestingly, callus cultures exhibited a higher antioxidant potential than in vitro cultures, suggesting that the method of cultivation may significantly influence the bioactivity of *Trifolium* species. The antioxidant potential of *T. pratense* callus cultures was further confirmed by Dyshlyuk et al.^51^ who additionaly reported that genistein exhibited the highest radical-scavenging activity among the tested isoflavonoids (biochanin A and ononin).

This finding may also explain the higher antioxidant potential observed in the leaf and flower extracts in our study, as these plant parts contained higher levels of genistein compared to callus cultures.

The skin-lightening activity of *T. rubens* extracts was also assessed using two complementary experimental models, involving mushroom tyrosinase and murine tyrosinase enzymes. Although overall inhibition was moderate, the observed effects were dependent on extract origin and enzyme source, indicating tissue-specific differences in bioactivity. Several studies have reported the ability of extracts from plants of the *Trifolium* genus to inhibit tyrosinase activity. However, data regarding tyrosinase inhibition by *T. rubens* extracts, as well as by callus cultures, are currently lacking. Previous research has demonstrated that the aerial parts of *T. nigrescens* Viv. exhibit tyrosinase inhibitory activity, and that flavonoid glycosides isolated from *T. nigrescens* subsp. *petrisavi* possess strong inhibitory properties against this enzyme^52^. Similarly, flavonoids identified in *T. echinatum* M. Bieb. have also been reported to show tyrosinase inhibitory activity^53^. Moreover, both in vitro and in vivo studies have confirmed that biochanin A isolated from *T. pratense* exhibits tyrosinase inhibitory effects, further supporting the role of *Trifolium*-derived flavonoids and isoflavonoids as potential natural tyrosinase inhibitors^54^.

There is a lack of studies investigating the effects of extracts from different *Trifolium* species, as well as their in vitro cultures, on elastase inhibition. The present study demonstrates that both flower extracts and callus culture extracts of *T. rubens* are capable of inhibiting elastase, indicating their potential relevance in anti-aging applications. A higher inhibitory effect associated with flower-derived material aligns with previous observations reported by Gościniak et al.^55^ who demonstrated stronger inhibition of collagenase—another metalloproteinase involved in skin firmness—by flower extracts of *T. pratense* compared with leaf extracts. These findings suggest that reproductive tissues of *Trifolium* species may be particularly enriched in compounds modulating enzymes implicated in skin aging.

Moreover, the present study did not confirm a cytotoxic effect on human keratinocytes (HaCaT) or the human malignant melanoma cell line A375. In contrast, studies by Borczak et al.^16^ demonstrated a reduction in the viability about 50% of A375 cells following treatment with *T. pratense* flower extracts, while no cytotoxic effect was observed on normal BJ fibroblast cells. Similarly, Šušaníková et al.^56^ also reported a low level of cytotoxicity of commercially purchased flower extracts toward NIH-3T3 mouse embryonic fibroblast and HaCaT keratinocytes.

Overall, multivariate analyses demonstrate that callus cultures exhibit a unique metabolic identity that cannot be inferred from differentiated plant organs, underscoring the importance of tissue-specific approaches when evaluating phytochemical composition and bioactivity. Callus cultures were primarily associated with isoflavones and tyrosinase inhibition, whereas leaf samples clustered with flavonols and elastase inhibition. These results indicate that biological activity patterns are tightly linked to tissue-dependent phenolic profiles. Given the limited and declining natural availability of this species, callus cultures may constitute a sustainable and ethically justified alternative to conventional plant material. Previous research has demonstrated that callus cultures can effectively replace whole plants, particularly in the case of rare or endangered taxa. Preliminary investigations of *T. rubens* callus cultures suggest their capacity to accumulate secondary metabolites exhibiting notable biological activities. In addition, extracts derived from these cultures have shown antioxidant activity and inhibitory effects against enzymes such as elastase and tyrosinase. Nevertheless, comprehensive studies are required to refine culture conditions, including the optimization of plant growth regulator composition, light regimes, and the application of elicitors and precursors, as well as the selection of appropriate culture systems. Such optimization may facilitate the future utilization of callus culture extracts in cosmetic and pharmaceutical applications, including skin-brightening products, anti-aging formulations, and agents aimed at reducing oxidative stress. Importantly, cytotoxicity assays conducted on HaCaT cells indicate that these extracts are safe for topical use. Previous studies indicate that callus cultures of the *Trifolium* genus subjected to optimization strategies, including culture condition optimization and the application of precursors and elicitors, may enhance isoflavonoid production as well as their biological properties, such as antioxidant activity. Ercetin et al.^27^demonstrated that the concentration of isoflavonoids in *T. pratense* callus cultures can be influenced by the type of plant growth regulators applied. Furthermore, Twaij et al.^30^ reported that the use of precursors and elicitors in in vitro cultures of *T. resupinatum* led to increased isoflavonoid production and a higher antioxidant potential of the obtained extracts. Additionally, Reis et al.^29^ showed that isoflavonoid content in in vitro cultures of *T. pratense* may depend on the duration of the cultivation period. Therefore, our findings indicate the potential for further research focused on the optimization of *T. rubens* callus cultures, as well as the implementation of various strategies aimed at enhancing the production of secondary metabolites in these cultures.

To sum up, this study provides the first comprehensive insight into the phytochemical composition and biological potential of *T. rubens* extracts obtained from leaves, flowers, and callus cultures. The results demonstrate that all tested extracts are rich in polyphenolic compounds, including flavonoids and isoflavonoids, which may contribute to their observed antioxidant and anti-aging properties. Notably, callus cultures exhibited more than twice the total isoflavonoid content compared to conventionally cultivated plant material, highlighting their potential as a sustainable source of bioactive compounds.

Among the identified metabolites, genistein—a key isoflavonoid with strong anti-aging and antioxidant effects—was successfully released from all extracts and penetrated the skin in ex vivo studies. Leaf extracts showed the highest genistein permeation and accumulation, suggesting their superior suitability for topical applications. Furthermore, all extracts demonstrated antioxidant activity and moderate inhibitory effects against elastase and tyrosinase, enzymes involved in skin aging and pigmentation. These effects may be associated with the presence of polyphenolic compounds. Importantly, cytotoxicity assays confirmed that the extracts are safe for use on human keratinocytes, supporting their potential inclusion in cosmetic formulations.

The findings emphasize the value of biotechnological approaches, such as callus culture, for producing rare and endangered plant species without compromising biodiversity. Future research should focus on optimizing in vitro cultures conditions and exploring strategies to enhance metabolite production, paving the way for the development of innovative cosmetic and dermatological products based on *T. rubens*.

## Materials and methods

### Parent plant material

Leaf, flower as well as the seeds of *T. rubens* (Fig. 1a) were collected in July 2024 from the Botanical Garden in Kielce (50°51’59.20"N, 20°35’56.33"E, 280 m a.sl.). The *T. rubens* seedlings were originally introduced into the garden’s collection of rare and endangered species from their natural habitat in the Małopolska Upland, in Pęczelice (Pińczów District), where the species grows on xerothermic grasslands (50°26’35.49"N, 20°47’07.55"E, 258 m a.s.l.). The plant species was identified by Prof. Renata Piwowarczyk. The seeds were stored at the Center for Research and Conservation of Biodiversity (Institute of Biology, Jan Kochanowski University, Uniwersytecka 7, 25–406 Kielce, Poland; 50.882032, 20.657719). Prior to analysis, the plant material was lyophilized using a Labconco freeze dryer (Labconco Corporation, Kansas City, MO, USA). Reference specimens were deposited in the Herbarium of Jan Kochanowski University in Kielce (KTC), located at the Institute of Biology, Uniwersytecka 7, 25–406 Kielce, Poland (50.882032, 20.657719). Although the collection does not have numerical identifiers, species are organized alphabetically in boxes labeled with their respective species names, along with a label indicating the collection site and the material used for analysis.

### **Initiation of** in vitro **cultures**

The seeds of *T. rubens* were surface-sterilized with 0.1% (v/v) chloramine T for 10 min while being continuously stirred, followed by three rinses with sterile distilled water. Sterilized seeds were then inoculated onto Murashige and Skoog (MS) medium^57^ supplemented with 0.8% (w/v) plant agar (Duchefa, Netherlands), 3% (w/v) sucrose, 1 mg/L BA, 0.5 mg/L NAA, and 0.25 mg/L GA₃ (pH = 5.8). After four weeks, a shoot was formed, which over next four weeks transformed into a callus. The in vitro cultures were maintained at 25±2°C under continuous white artificial LED illumination with an intensity of 1 W/m² (Heckermann, Poland) and subcultured every 20 days.

### **Experimental** in vitro **cultures**

The experiment included the cultivation of *T. rubens* callus on agar medium (Fig. 1b). Callus cultures were maintained on standard MS medium supplemented with 0.8% (w/v) plant agar (Duchefa, Netherlands), 3% (w/v) sucrose, 1 mg/L BA, 0.5 mg/L NAA, and 0.25 mg/L GA₃. The cultures were incubated under continuous white light (1 W/m²) at a temperature of 25±2°C, with each growth cycle lasting 20 days (3 series were performed).

### Extraction

Lyophilized (Labconco Corporation, Kansas City, MO, USA) and pulverized biomass from leaf, flower and callus cultures was extracted in 70% ethanol (v/v) (ChemLand, Piekary Śląskie, Poland). The selection of 70% ethanol was based on its effectiveness in extracting a broad range of both polar and moderately non-polar bioactive compounds, including phenolics and flavonoids, while maintaining low toxicity and compatibility with biological assays. Extraction was performed using an ultrasonic bath CD-482 (Hotair, Zawiercie, Poland) (30 min, room temperature). For biological assays 1% extracts were evaporated at 45 °C (Concentratorplus/Vacufuge^®^ plus, Eppendorf, Hamburg, Germany) and stored at − 80 °C. For skin penetration studies, 5% extracts were prepared by ultrasonic extraction of dried plant material in 70% ethanol, filtered (Whatman filter paper No. 10.), and stored at 4°C in the dark until use.

### Polyphenol profiling

The determination of polyphenol concentration was carried out using the HPLC-DAD method, following the procedure outlined in earlier studies^58,59^. Ethanolic extracts were subjected to chromatographic analysis using an HPLC-DAD system (Merck-Hitachi, Merck KGaA, Darmstadt, Germany) equipped with a Purospher RP-18e analytical column (4 × 250 mm, 5 μm; Merck, Boston, MA, USA). Separation was performed with two mobile phases: phase A consisting of methanol and 0.5% acetic acid (1:4, v/v), and phase B composed of methanol. The gradient elution program was as follows: 0–20 min, 0% B; 20–35 min, 0–20% B; 35–45 min, 20–30% B; 45–55 min, 30–40% B; 55–60 min, 40–50% B; 60–65 min, 50–75% B; and 65–70 min, 75–100% B, followed by a 15-minute hold time. Chromatographic conditions were maintained at 25°C with a flow rate of 1 mL/min, an injection volume of 10 µL, and detection at 254 nm.

Qualitative and quantitative analyses were conducted by comparing retention times and UV spectra with those of authentic standards. The reference compounds included 29 phenolic acids: caffeic, 4-*O*-caffeoylquinic, caftaric, chlorogenic, *m*-, *o*-, and *p*-coumaric, cryptochlorogenic, 1,5-, 3,4-, and 3,5-dicaffeoylquinic, 3,4-dihydroxyphenylacetic, ellagic, ferulic, 4-feruloylquinic (y = 21290104.75x-474737.80, R^2^ = 0.9992), gallic, gentisic, hydrocaffeic, *p*-hydroxybenzoic, isochlorogenic, isoferulic, neochlorogenic, phenylacetic, protocatechuic, rosmarinic, salicylic, sinapic, syringic, and vanillic acids—as well as their precursors (benzoic and cinnamic acids). Additionally, 24 flavonoid standards were used: apigenin (y = 59476721.14x-1168407, R^2^ = 0.9995), apigenin-7-glucuronide, astragalin, avicularin, cymaroside, chrysin, guaiaverin, hyperoside, isoquercetin, kaempferol (y = 59797159.34x-3146083.29, R^2^ = 0.9996), kaempferol-4-glucoside, kaempferol-7-rhamnoside, quercetin, quercimetrin, quercitrin, luteolin, myricetin (y = 44643051.91x-1581300.73, R^2^ = 0.9996), naringin, populin, rhamnetin, robinin, rutin, trifolin (y = 80650805.89x-583711.04, R^2^ = 0.9997), vitexin and also 14 isoflavones: biochanin A (y=261439193x + 12000558.50, R^2^ = 0.9995), calycosin (y = 199436965.90x + 797821.46), R^2^ = 0.9994), calycosin 7-*O*-glucoside (y = 117792540.60x + 655916.47, R^2^ = 0.9992), daidzein (y = 255563607.50x + 1361006.04, R^2^ = 0.9994), formonoetin (y = 208694124.80x + 783313, R^2^ = 0.9999), genistein (y = 134155153.20x-52607.13, R^2^ = 0.9996), genistin, glycitin, hesperetin, hesperidin, ononin, pseudobaptigenin (y = 150867549.80x + 311503.14, R^2^ = 0.9999), rothindin, taxifolin. All standards were purchased from Sigma-Aldrich (Saint Louis, MO, USA). Quantification used external calibration curves. According to the validated protocol^58^, limits of detection (LOD) ranged from 0.007 to 0.024 mg/mL, and limits of quantification (LOQ) from 0.023 to 0.072 mg/mL.

## Ex vivo **permeation experiments**

### Ex vivo skin permeation of genistein

In the ex vivo permeation experiments, porcine abdomen skin was used because of its similar permeability to human skin^60,61^. The skin for the experiment was prepared and stored as described previously^62,63^. The skin was obtained from a local slaughterhouse. First, fresh skin was washed several times in PBS buffer pH 7.4 and skin fragments of 0.5 mm thickness was subsequently dermatomized. Next, it was divided into 2 cm × 2 cm pieces, wrapped in aluminium foil and stored in a freezer at −20°C until use. Skin samples were stored for no longer than three months. On the day of the experiment, skin samples were slowly thawed at room temperature for 30 min.

Permeation experiments were conducted using Franz diffusion cells (SES GmbH Analyse Systeme, Bechenheim, Germany) with a diffusion area of 1 cm^2^. The acceptor chamber was filled with PBS solution (pH 7.4), and a constant temperature of 37.0°C ± 0.5°C was maintained in each diffusion unit. The content of the acceptor chamber was stirred with a magnetic stirring bar at a consistent speed across all cells. The donor chamber had a volume of 2 mL, while the acceptor chamber had a volume of 8 mL.

Undamaged pieces of skin were placed between the donor and acceptor chambers of the Franz diffusion cells. Skin integrity was measured using an LCR meter 4080 (Voltcraft LCR 4080; Conrad Electronic, Germany) according to previous studies^63^. Next, 1 mL of each ethanolic extract of *T. rubens* applied to the outer side of the skin in the donor compartment. The penetration study was carried out over 24 h. At 1, 2, 3, 5, 8 and 24 h, 0.5 mL samples were withdrawn from the acceptor chamber and replaced with the same volume of fresh PBS. The concentrations of phenolic acids in the acceptor phase were evaluated using HPLC. The cumulative mass (µg) of genistein was calculated based on the obtained concentrations.

### HPLC analysis of genistein

The identification of genistein in the acceptor fluid after permeation as well as fluid after skin extraction based on the method previously described by Kika et al.^64^. Used were carried out using an HPLC system from Knauer (Berlin, Germany) coupled with the WellChrom UV K-2600 detector. The tested components were separated on a 125 × 4 mm column containing Hypersil ODS C18 with a particle size of 5 μm. The detection of genistein was performed by UV absorption at λ = 278 nm. The compound was identified based on its retention time and by comparison with standard under the same conditions. The mobile phase consisted of 1% aqueous acetic acid solution (A) and 100% MeOH (B). The samples were eluted with the following gradient: 90% A and 10% B from 0 to 6 min, 84% A and 16% B from 7 to 25 min, 72% A and 28% B from 26 to 37 min, 65% A and 35% B from 38 to 47 min, 50% A and 50% B from 48 to 64 min, and 90% A and 10% B from 65 to 70 min, to restore the initial conditions, before injection of a new sample. The flow rate was 0.8 mL/min, and the injection volume was 20 µL. All samples were analysed three times.

### Antioxidant activity of the acceptor and the skin extraction fluids

The scavenging activity of stable DPPH free radicals in the acceptor fluid and skin extraction fluid was determined as previously described^65^. In brief, 0.15 cm³ of each tested sample was combined with 2.85 cm³ of a 0.3 mM DPPH solution prepared in 96% (v/v) ethanol. The absorbance of the DPPH working solution was standardized to 1.00 ± 0.02 at 517 nm using 70% (v/v) ethanol as a reference. After 10 min of incubation in the dark at room temperature, absorbance was recorded at 517 nm with a Hitachi UV-Vis U-5100 spectrophotometer. Each extract was analyzed in triplicate to ensure reproducibility.

The method for assessing the ABTS radical scavenging activity of the acceptor fluid and skin extraction fluid was previously described^65^. In brief, a 7 mM ABTS solution was prepared in 2.45 mM aqueous potassium persulfate and used as the stock solution. After mixing the reagents, the solution was kept in the dark at room temperature for 24 h to allow radical formation. It was then diluted with 50% (v/v) methanol to obtain a working solution with an absorbance of 1.00 ± 0.02 at 734 nm.

To determine antioxidant activity, 2.5 cm³ of the ABTS working solution was mixed with 0.025 cm³ of the plant extract in a spectrophotometric cuvette. Following a 6-minute incubation at room temperature, the absorbance was recorded at 734 nm. Each extract was analyzed in triplicate.

The radical scavenging activity (RSA) for both DPPH and ABTS radicals was calculated as the percentage of inhibition using the equation:

$\:\mathrm{\%}\mathrm{RSA}=(1-\frac{{A}_{s}}{{A}_{c}})\times\:100\mathrm{\%}$where $\:{A}_{s}$is the absorbance of the tested sample and $\:{A}_{c}$is the absorbance of the control. All measurements were performed in triplicate for each extract.

### Assessment of biological activities

#### DPPH radical scavenging assay

The antioxidant capacity of the extracts was evaluated following the procedure described by Matejić et al., with subsequent modifications^66^. A DPPH solution was prepared by dissolving 9.8 mg of DPPH in 50 mL of ethanol and stirring it on a magnetic stirrer for one hour in the dark. Subsequently, 25 mL of this solution was transferred to a 100 mL volumetric flask and made up to volume with ethanol. The absorbance of the solution measured at λ = 540 nm using a cuvette UV-Vis spectrophotometer (DR6000, Hach, Poland) was ca. 1. The tested extracts were dissolved in DMSO to a concentration of 2 mg/mL and serially diluted in a 96-well plate, followed by the addition of 100 µL of DPPH solution to each well. The mixtures were then incubated in the dark for 10 min. Each sample was analyzed in triplicate. Background samples consisted of 100 µL of extract and 100 µL of the solvent, while the control contained 100 µL of the solvent and 100 µL of DPPH solution. The same procedure was applied to vitamin C, which served as a reference compound with well-established antioxidant activity. All measurements were carried out using a microplate spectrophotometer (FilterMax F5, Molecular Devices, CA, USA).

The percentage of DPPH radical scavenging was calculated using the following equation:

$$\:\mathrm{\%}\:\mathrm{D}\mathrm{P}\mathrm{P}\mathrm{H}\:\mathrm{s}\mathrm{c}\mathrm{a}\mathrm{v}\mathrm{e}\mathrm{n}\mathrm{g}\mathrm{i}\mathrm{n}\mathrm{g}=[1-\frac{\mathrm{Abs(S)}}{\mathrm{Abs(C)}}]\times\:100$$

where: Abs(S)—the absorbance of the sample, Abs(C)—the absorbance of the control sample.

### ABTS radical scavenging assay

The antioxidant activity based on ABTS free radical neutralization was evaluated according to the method described by Re et al., with minor modifications^67^. In brief, 0.96 g of ABTS was dissolved in 250 mL of a 2.45 mM potassium persulfate (K₂S₂O₈) solution. The absorbance of the resulting mixture was adjusted using a cuvette spectrophotometer (DR6000, Hach, Poland) to approximately A₄₀₅≈1. The extracts were serially diluted in DMSO, starting from a stock concentration of 10 mg/mL. For the assay, 15 µL of each extract was transferred to a 96-well plate (in triplicate), followed by the addition of 135 µL of the ABTS solution. The mixtures were incubated in the dark for 15 min. Background wells contained 15 µL of extract and 135 µL of solvent, while the control consisted of 15 µL of solvent and 135 µL of ABTS solution. Vitamin C was analyzed under identical conditions as a reference antioxidant. All absorbance measurements were performed at 405 nm using a plate spectrophotometer (FilterMax F5, Molecular Devices, CA, USA). The percentage of ABTS radical scavenging activity was calculated according to the following equation:

$$\:\mathrm{\%}\:\mathrm{ABTS\:scavenging}=[1-\frac{\mathrm{Abs(S)}}{\mathrm{Abs(C)}}]\times\:100$$

where Abs(S) is the absorbance of the sample and Abs(C) is the absorbance of the control.

#### Tyrosinase inhibitory assay

The test was performed following the procedure described by Uchida R. and co-authors with further modifications, using commercially available mushroom tyrosinase (Sigma Aldrich, St. Louis, MO, USA) and murine tyrosinase present in the lysate of murine melanoma cell B16F10 (ATCC CRL-6475; LGC Standards, Łomianki, Poland)^68^. For both assays extracts were diluted in phosphate buffer (100 mM, pH = 6.8) to concentrations of 1 mg/mL, 0.5 mg/mL, 0.25 mg/mL. The reference tyrosinase inhibitor was kojic acid prepared at the same concentrations. For the mushroom tyrosinase inhibitory assay, 20 µL of samples were applied onto a 96-well plate in triplicate, 120 µL of 100 mM phosphate buffer pH 6.8 and 20 µL of fungal tyrosinase solution (500 U/mL) were added. After 10 min of incubation at room temperature, 40 µL of L-DOPA (4 mM, Sigma Aldrich, St. Louis, MO, USA) was added. The plate was incubated for 20 min at room temperature. For the murine tyrosinase inhibitory assay, 20 µL of samples were mixed with 40 µL 4 mM L-DOPA and 100 mM phosphate buffer pH 6.8 with a volume of B16F10 lysate containing 20 µg protein. The plate was incubated for 4 h at 37 °C. The background for both assays was 20 µL of extract and 180 µL of phosphate buffer. The control sample (equal to 100% tyrosinase activity) was carried out the same as extracts with modification - the solvent instead of the extract. After incubation time, the absorbance at λ = 450 nm was measured using a plate spectrophotometer (FilterMax F5 Molecular Devices).

### Elastase inhibitory assay

The inhibition of elastase (Sigma Aldrich, St. Louis, MO, USA) was established using the protocol described by Horng and co-workers with further modifications^69^. The TRIS-HCL buffer was prepared by dissolving 15.76 mg of TRIS-HCL in 100 mL ultrapure water and adjusted to a pH value of 7.8. Extracts were diluted in TRIS-HCL buffer to concentrations of 1 mg/mL, 0.5 mg/mL, 0.25 mg/mL. 15 µL of diluted extracts were mixed with 90 µL TRIS-HCL buffer (pH = 7.8) and 25 µL elastase working solution (0.05 mg/mL, 0.2 U, in TRIS-HCL buffer). Following 10 min incubation at room temperature, 20 µL of 2.9 mM SANA (N-succ-(Ala)−3-nitroanilide) (Sigma Aldrich, St. Louis, MO, USA) was added to each sample, and incubated for 20 min at 37 °C. The background was 15 µL of each extract in each concentration mixed with 25 µL elastase working solution (0.05 mg/mL, 0,2 U, in TRIS-HCL buffer) and 110 µL TRIS-HCL buffer (pH = 7.8). The control sample showing 100% elastase activity was prepared by adding 105 µL TRIS-HCL buffer (pH = 7.8) to 25 µL elastase working solution (0.05 mg/mL, 0.2 U, in TRIS-HCl buffer) and 20 µL of 2.9 mM SANA. The reference sample was epigallocatechin (Sigma Aldrich, St. Louis, MO, USA) tested in exactly the same way as the extracts. The absorbance of all samples at λ = 405 nm was measured using a FilterMax F5 microplate reader (Molecular Devices). The activity of elastase was calculated based on the equation:

% of elastase activity = [Abs(S)/Abs(C)] × 100%, where: Abs(S)—the absorbance of the sample, Abs(C)—the absorbance of the control sample.

#### *In vitro cytotoxicity*

The cytotoxic effect was analyzed on HaCaT keratinocytes (CLS Cell Lines Service GmbH, Eppelheim, Germany)^70^ and human malignant melanoma cell line A375 (LGC Standards, Łomianki, Poland) using the neutral red uptake test, according to the procedure described previously^71^. HaCaT and A375 cells were cultivated in DMEM (Dulbecco′s Modified Eagle′s Medium, Sigma Aldrich, St. Louis, MO, USA), supplemented with 10% (*v/v*) FBS (PAN-Biotech GmbH, Aidenbach, Germany) at 37 °C in a humidified atmosphere with 5% CO_2_. The cells were plated onto 96-well plates (1 × 10^4^ cells/well). After 24 h incubation, extracts were applied to the cells for 48 h in 5 concentrations starting from 0.2 mg/mL. Control cells were grown in conditioned medium supplemented with equal volume of the extract solvent (DMSO). The neutral red solution (33 µg/mL) was diluted in DMEM supplemented with 1% (*v/v*) FBS and was incubated with the cells for 2 h. The cells were then washed with DPBS (Dulbecco′s Phosphate Buffered Saline, Sigma Aldrich, St. Louis, MO, USA) and lysed in an acidified ethanol solution (50% *v/v* ethanol, 1% *v/v* acetic acid, and 49% H_2_O). The absorbance of the released neutral red was measured at λ = 540 nm using a FilterMax F5 microplate reader (Molecular Devices, CA, USA). The mean absorbance value of the control cells was set at 100% viability and used to calculate the percentage of cellular viability following extract treatment. The experiment was performed in three separate attempts, with five repetitions per one attempt.

### Statistical analysis

All experiments were performed in triplicate, and results are expressed as mean ± standard deviation (SD). Prior to statistical analysis, data were tested for normality (Shapiro–Wilk test) and homogeneity of variances (Levene’s test). A one-way analysis of variance (ANOVA) was used. The significance of differences between individual groups was evaluated with Tukey’s test (α < 0.05). Statistical calculations were done using Statistica 13 PL software (StatSoft, Kraków, Poland). The Principal Component Analysis (PCA) was performed using PAST 4.03^72^ software to identify patterns and relationships between variables. Cluster analysis was conducted using hierarchical clustering with Euclidean distance and Ward’s method. The results were visualized as dendrograms combined with heatmaps using the Heatmapper tool ^73^.

## Data Availability

The authors declare that the data supporting the findings of this study are available within the paper. Should any raw data files be needed in another format they are available from the corresponding author upon reasonable request. Source data are provided with this paper.
